# Identification challenges from catastrophic aviation disasters: A forensic case series from 2 military air crashes

**DOI:** 10.1097/MD.0000000000044326

**Published:** 2025-09-12

**Authors:** Stefan Pricop, Miruna Cristian, Radu Adrian Nitu, Sorin Deacu

**Affiliations:** a Department of Forensic Medicine, County Clinical Emergency Hospital of Constanta, Constanta, Romania; b Faculty of Medicine, Ovidius University, Constanta, Romania; c Center for Research and Development of the Morphological and Genetic Studies of Malignant Pathology – CEDMOG, “Ovidius” University of Constanța, Romania; d Department of Cardiovascular Surgery, County Clinical Emergency Hospital of Constanta, Constanta, Romania.

**Keywords:** aviation disaster, carbonization injuries, forensic investigation, forensic pathology, identification methods, toxicology analysis

## Abstract

**Rationale:**

Accurate identification of victims in postcrash scenarios presents significant forensic challenges, particularly when the remains are extensively burned, fragmented, or carbonized. This study focused on multiple victims of 2 aircraft crashes, highlighting the intricate methods and obstacles encountered during their identification.

**Patient concerns:**

Severe thermal damage, widespread tissue degradation, and advanced carbonization have hindered traditional identification techniques, requiring a multidisciplinary forensic approach that combines DNA analysis, dental profiling, tattoo recognition, and artifact examination.

**Diagnoses:**

In addition to establishing the causes of death, which also required histopathological examinations, the difficulty involved in assigning the identity of each corpse made the forensic pathologists resort to all the information and details they could observe during the autopsy.

**Interventions:**

Personal objects such as jewelry, tattoos, and prosthetic devices helped to reconstruct the identity of each corpse, in some cases being assigned identification by exclusion.

**Outcomes:**

Severe cranial fractures, rib and thoracic injuries, and extensive organ damage, such as pulmonary contusions, alveolar hemorrhages, and hepatic lacerations, were consistently observed in all cases. Forensic dental identification, which, in the present case, helped identify one of the victims through dental analysis.

**Lessons:**

This study highlights the critical role of the identification of victims in circumstances in which the body is largely destroyed and underlines the importance and necessity of forensic identification through dental identification methods, which potentially represent one of the most effective methods that retain their accuracy regardless of the degree of destruction of the corpse.

## 1. Introduction

The investigation of aviation accidents is particularly complex because of the high impact forces typically involved and frequent occurrence of postcrash fires.^[1]^ A particularly tragic case, reported in Eastern Europe in recent years, involved the consecutive loss of 2 military aircraft (including a MiG aircraft) during routine operation and the subsequent rescue mission. Both incidents resulted in multiple fatalities among service personnel.^[2,3]^

Forensic examinations constitute an important step towards establishing the circumstances of death in such high impact disasters.^[4]^ Forensic pathology aids not only in identifying the victims and finding the cause of death but also makes it possible to identify the pattern of injuries, the time when the injury was inflicted, and the response of the person to the injury.^[5]^ The cases presented here reflect the meticulous work conducted by forensic specialists at the Clinical County Service of Forensic Medicine, where detailed postmortem and histological examinations of the victims were performed.

This article describes the difficulties caused by trauma in identifying disaster victims. By observing the general features noted between cases, this research hopes to provide invaluable feedback on how certain injuries that lead to mortality in aviation accidents are sustained.^[6]^ The results not only extend the knowledge base on these events, but also emphasize the importance of more attention being given to aspects such as safety regulations, advancement of forensic methods, and improvement of responses to disasters.^[7]^ Thus, the forensic perspective is a vital element of aviation safety and noise evidence.

## 2. Materials and methods

### 2.1. Methodology

Forensic investigations of aircraft accidents have been conducted at the Clinical County Service of Forensic Medicine. Each of the 8 cases underwent comprehensive forensic autopsies aimed at identifying the victims, determining the manner and cause of death, and distinguishing injury patterns and their connection to crash dynamics.

### 2.2. Case documentation

The medical experts were able to proceed with in-depth forensic investigations thanks to the comprehensive recording of the crash site together with the circumstances surrounding the recovery of victims. The details of high-energy collisions and postcrash fires were also incorporated into the analysis. Documentation from the crash sites was provided by the Service of Criminal Investigation and Criminology Department.

### 2.3. External and internal examination

External and internal examinations of the bodies were conducted in accordance with the National Forensic Institute guidelines. All externally relevant elements were documented, including height, traumatic injuries, scars, tattoos, distinctive constitutional or facial features, personal belongings and clothing, overall nutritional status, and postmortem changes. The total body surface area affected by burns was evaluated using “the rule of nines.” The internal examination was adapted to each individual case as extensive destruction precluded the standard autopsy protocol. The assessment included the evaluation of internal organs, both externally and in cross-section, along with bones and soft tissues, with detailed documentation of any observed abnormalities. The extent of carbonization and soft tissue loss was noted to indicate postmortem and perimortem effects. Emphasis was placed on ruptured or lacerated tissues with associated bleeding due to severe trauma.

### 2.4. Histological analysis

All fragments collected during the autopsies were processed in the Pathology Department using standardized protocols: fixation for 24 to 48 hours in 10% formaldehyde, after which the fragments were processed with a Bio-Optika automated tissue processor. The paraffin-embedded blocks obtained were then sliced at 5 µm and stained with hematoxylin–eosin (HE).

### 2.5. Toxicological testing

Blood and other biological samples (kidney and liver) were tested to check for blood alcohol, carbon monoxide, and other toxic substances.

### 2.6. Identification procedures

Owing to extensive carbonization, an identification protocol similar to Interpol’s Disaster Victim Identification (DVI) system (based on dental records, DNA (deoxyribonucleic acid) analysis, and other biological markers) was utilized to successfully identify the remains. This consists of 5 key phases.

#### 2.6.1. Phase 1: scene investigation

The first phase consisted in securing and processing the disaster site by the authorities. Human remains and associated evidence were recovered and documented. The remains were labeled appropriately in plastic bags. Standardized procedures ensure the preservation of forensic integrity, prioritizing safety and proper evidence collection. This phase establishes a foundation for subsequent identification.

#### 2.6.2. Phase 2: postmortem (PM) examination

The recovered remains were then transported to a designated mortuary for a detailed forensic examination. This phase included DNA sampling, forensic odontology, and other methodologies to obtain individualizing features. Personal effects were also analyzed to gather potentially useful data. Not all identification methods were employed, as most cases were successfully identified using only 1 or 2 parameters, such as tattoos or personal effects.

#### 2.6.3. Phase 3. antemortem (AM) data collection

Simultaneously, data were collected from family members, as well as dental and medical records (where available). Interviews were also conducted to gather relevant information regarding the physical attributes and unique characteristics (tattoos, scars) of the victims.

#### 2.6.4. Phase 4: reconciliation

Forensic teams cross-referenced postmortem findings with antemortem data to establish a preliminary positive identification. Criminologists and forensic pathologists reviewed the evidence together and confirmed the identities by correlating the PM and AM data.

#### 2.6.5. Phase 5: review and documentation

This last phase involves reviewing the identification process, ensuring adherence to forensic and legal standards and addressing potential issues.

### 2.7. DNA testing

DNA analysis was outsourced to the National Institute of Criminology, which subsequently provided the results. Consequently, detailed information regarding the specific methodologies employed at different institutions in the testing process was not available to the authors.

### 2.8. Time of death estimation

The classical postmortem indicators, livor mortis, rigor mortis, and algor mortis, could not be accurately assessed because of extensive destruction of the bodies. In addition, no signs of decomposition or insect activity were observed. Histological examination of the available organ fragments revealed varying degrees and patterns of hemorrhage, anemia, and contusions, which are findings suggestive of antemortem trauma. Based on these findings, correlated with circumstantial data obtained from the scene investigation – including the known time of the crash and the sequence of events surrounding the accidents – it was determined that the victims were alive at the known moment of impact.

## 3. Results

### 3.1. On-site recovery and initial handling of bodies

At the crash site, bodies and detached body segments were collected and sealed in black plastic bags and labeled B to L. The remains of the MiG captain, including several body fragments, were also recovered from the aircraft. The bodies were randomly assigned to forensic pathologists, who numbered each victim from 1 to 8, including the captain (Victim 8). Toxicological and serological (standard ABO system) tests were conducted on blood samples from the bodies, as well as liver and kidney specimens, in cases where blood was not available. DNA samples from relatives were obtained through buccal swabs, while DNA samples from victims were taken from various tissue specimens (muscle, bone marrow) and, where available, blood samples. Interviews were also conducted with the victims’ family members to gather relevant information for the identification process, such as prior medical procedures, distinctive marks (tattoos, scars), and details regarding commonly worn clothing and accessories.

### 3.2. Victim 1 – Bag H: identification via external features and tattoos

Bag H contained a body with partial burns on the lower limbs, along with fractures and soft tissue destruction but without facial disfigurement, allowing for a straightforward identification. Additionally, a tattoo was observed – a distinctive mark that can serve as an identifying feature unless extensive body destruction through burns or carbonization occurs. The cause of death was crushing of the body. Toxicological tests performed on blood samples, including carboxyhemoglobin, were negative. The contents of Bags I and J (muscle tissue) were assigned to the body in Bag H based on DNA testing, which was also cross-checked with the maternal DNA samples. Serological analysis of blood samples identified the blood group as type A.

### 3.3. Victim 2 – Bag D: DNA-based identification and cause of death

Bag D contained a completely carbonized body (100% body surface), and the final identification was based exclusively on DNA testing. Two potential identities remained after excluding others. Biological samples from relatives were compared with those from the body in Bag D. Serological analysis of the blood samples identified the blood group as type A; however, the remaining 2 individuals under consideration had the same blood type, making DNA testing the only definitive identification method. The cause of death was trauma and hemorrhagic shock, with postmortem carbonization. Toxicological tests were negative for alcohol, carboxyhemoglobin, and other psychoactive substances.

### 3.4. Victim 3 – Bag E: carbonized body and identification via dental prosthesis

Bag E contained a body that was completely carbonized (100% of the body surface), thus rendering external identification impossible. Forensic dental identification, including dental charting, was performed. A dental prosthesis was found and analyzed. It was identified as a partial acrylic-metal prosthesis documented in the medical records of one of the known military personnel onboard the helicopter. DNA testing was conducted by comparing the relative’s DNA with the DNA of the body, confirming its identity. Additionally, 2 bags labeled C and K containing body segments – a left forearm and a left foot – were assigned to the body in Bag E based on DNA analysis comparing samples taken from the bodies during autopsy with relatives’ DNA. Due to the high degree of destruction and absence of blood, blood type determination, blood alcohol concentration, and carboxyhemoglobin levels could not be established. Toxicological tests performed on the collected organ fragments (liver and kidney) yielded negative results.

### 3.5. Victim 4 – Bag L: severe burns and identification via military ID tag

Bag L contained a body with third- and fourth-degree burns covering approximately 80% to 90% of the total body surface, with significant craniofacial destruction. The cause of death was thoracic trauma with rib fractures, pleuropulmonary rupture, aortic rupture, and myocardial laceration. Burns were deemed postmortem, given the absence of carboxyhemoglobin in the blood. Toxicological test results were negative for alcohol and psychoactive substances. The initial identification was aided by a military identification tag found around the body’s neck, which was inscribed with a name. The presumed identity was confirmed by DNA testing, which compared samples taken from the body with those from the victim’s father. Serological analysis of blood samples identified the blood group as type A.

### 3.6. Victim 5 – Bag F: DNA-based identification and cause of death

Bag F presented identification challenges similar to those of Bag D, lacking distinctive identification features. Among the 2 remaining unidentified individuals at the time of the forensic autopsy, DNA test results were awaited for final identification. The cause of death was determined to be carboxyhemoglobin intoxication and burns with extensive body destruction. Toxicological tests were positive only for carboxyhemoglobin. Serological analysis of blood samples identified the blood group as type A.

### 3.7. Victim 6 – Bag B: identification and findings

The body in Bag B was identified during the forensic autopsy via external examination, as the craniofacial region remained intact, facilitated by the presence of an intact uniform and wristband on the left wrist. Biological samples were collected for toxicological testing, which yielded negative results for alcohol, psychoactive substances, and carboxyhemoglobin. The cause of death was craniocerebral trauma, with postmortem burns affecting the lower limbs. DNA analysis confirmed the identity of the deceased. Serological analysis of the blood samples identified the blood group as type O.

### 3.8. Victim 7 – Bag G: identification via wedding ring and DNA testing

Bag G contained a body that was entirely carbonized (100% of body surface). The presumed identity was initially based on a wedding ring which was identified at the external examination of the body and recognized by the deceased’s spouse. DNA testing confirmed this identity. The cause of death was craniocerebral trauma with hemorrhage and cerebral contusion. Toxicological tests were negative for alcohol, carboxyhemoglobin, and psychoactive substances. Paracetamol and a metabolite indicative of prior analgesic and antipyretic medication use were detected. Serological analysis of the blood samples identified the blood group as type O.

### 3.9. Victim 8 – forensic autopsy of the MiG captain

The forensic autopsy of the MiG captain did not require multiple identification methods, as he was the sole occupant of the aircraft. Severe body crushing with postmortem carbonization was the cause of death. Several body fragments were retrieved from the aircraft and subjected to DNA analysis to confirm their identity. DNA samples collected from the father of the victim were used for comparison. Due to the extensive destruction of the body, biological samples could not be obtained for toxicological or serological examinations.

Table 1 provides a detailed overview of the identification challenges faced during the examination of the 8 victims with varying degrees of trauma and burn injuries. Each case presented unique obstacles, ranging from severe carbonization to partial disintegration of the remains. The table outlines the methods utilized to overcome these barriers, such as DNA analysis, dental record matching, and personal artifact identification, as well as the results of the blood group determination.

**Table 1:** Identification challenges.

Victim ID	Challenges faced	Blood group	Identification methods used
1st victim	Attributing scattered body fragments to the correct individual.	A	Tattoo analysis, DNA analysis, visual identification
2nd victim	Extensive thermal damage obscures distinguishing physical features, making visual identification unlikely.	A	DNA analysis
3rd victim	Completely carbonized body (100% body surface) with limb disarticulation and partial body recovery hinder complete physical profiling; reliance on dental structures and DNA.	Unavailable sample	Dental analysis (intact structures visible), DNA analysis
4th victim	High degree of burns obscures physical features, but identification tag provides immediate potential for verification.	A	Tag-based identification, cross-referenced with military records; DNA analysis
5th victim	Severe carbonization limits physical feature identification.	A	DNA analysis
6th victim	Craniofacial region remained intact.	O	Visual identification, uniform analysis, wristband verification, DNA testing
7th victim	Advanced burn damage obscures physical features; ring provides personal information; jewellery provides a potential clue but requires database cross-referencing DNA may be limited.	O	Wedding ring analysis, DNA analysis
8th victim MiG Captain	Severe thermal injuries and fragmentation obscure features; reliance on ID tag and DNA for verification.	Unavailable sample	ID tag analysis, cross-referencing with military records, DNA analysis

Images corresponding to each victim listed in Table 1 are provided in a Supplementary Material, available from the corresponding author on reasonable request. Each victim is represented by detailed images that illustrate key pathological features including tissue alterations, cellular damage, and specific markers of injury.

Below, we present the profound structural damage to the skin following exposure to intense thermal injury, highlighting the characteristic features of epidermal necrosis and carbonization. The slides were examined under an Accuscope EXC-500 light microscope, and representative photos were acquired on a computer using an Excelis HD video camera.

**Figure 1.**
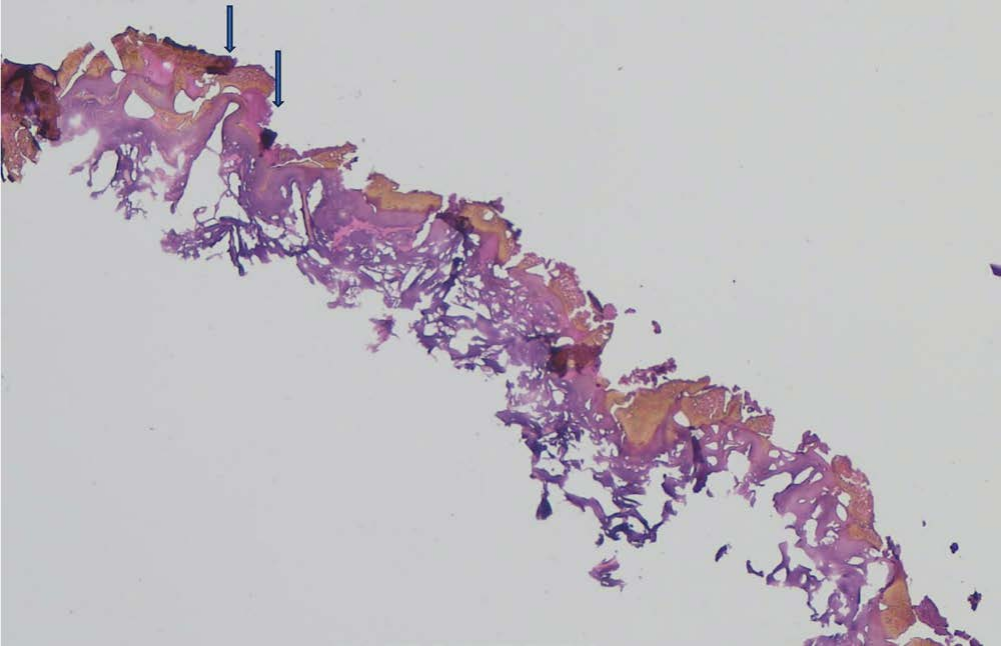
Total epidermal necrosis with the presence of soot particles on its surface (arrows), accompanied by detachment from the underlying dermis, indicative of carbonization (hematoxylin–eosin stain, 10× magnification).

Figure 1 shows complete necrosis of the epidermis, characterized by the presence of soot particles adhering to its surface, suggesting exposure to intense thermal or fire-related injury. The epidermis is entirely detached from the underlying dermis, a process indicative of carbonization. This separation highlights the structural damage caused by extreme heat, resulting in disruption of the dermo-epidermal junction. The presence of soot particles further confirmed the exposure to combustion byproducts.

**Figure 2.**
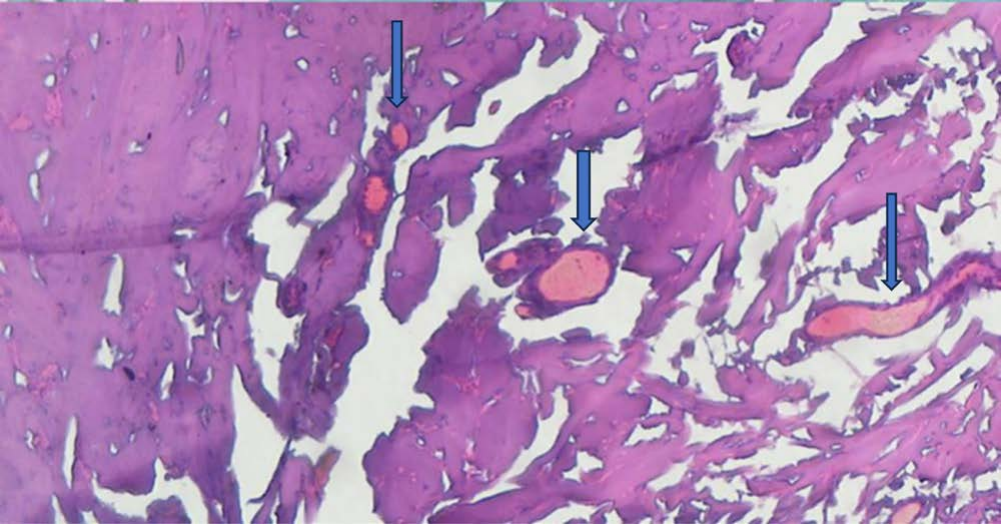
Dermal necrosis with the complete loss of skin appendages and the presence of blood vessels containing lysed erythrocytes (arrows), characteristic of a fourth-degree burn (hematoxylin–eosin stain, 10× magnification).

The histopathological examination in Figure 2 revealed extensive dermal necrosis, characterized by the complete destruction of skin appendages, including hair follicles, sebaceous glands, and sweat glands, indicating severe thermal injury. Blood vessels within the dermis show marked damage, with the presence of lysed erythrocytes, further emphasizing the extent of vascular compromise caused by the burns.

Table 2 emphasizes both the common patterns and unique aspects observed across the 8 victims. Severe cranial fractures, pulmonary contusions, and hepatic lacerations were sustained by the victims because of exposure to high-energy impacts during the plane crash. The postcrash fires were indeed severe and resulted in many cases of extensive thermal injuries, including burns that extended from third to fourth degree. However, unique findings provided critical insights into the individuality of each case, such as cervical vertebral disruption in victim 1, prominent ischemic kidney damage in victim 3, and subpleural emphysema with bronchial blood aspiration in victim 7.

**Table 2:** Comparison of common patterns and unique findings across forensic cases.

Aspect	Victim 1	Victim 2	Victim 3	Victim 4	Victim 5	Victim 6	Victim 7	Victim 8 – MiG
Common patterns	Severe cranial fractures; pulmonary contusions; hepatic subcapsular hematoma; cerebral hemorrhages.	Cranial fractures; bilateral pulmonary hemorrhage; hepatic rupture; extensive thoracic trauma.	Cranial fractures with brainstem hemorrhages; pulmonary alveolar hemorrhages; hepatic fragmentation.	Complete carbonization; severe thermal injuries; organ obliteration.	Thoracic fractures with pulmonary contusions; massive hepatic rupture; splenic laceration.	Cranial fractures; pulmonary contusions; renal ischemic necrosis.	Cranial fractures extending to the skull base; bilateral pulmonary ruptures; hepatic laceration.	Severe cranial fractures; fragmented remains with multiple fractures of the long bones; extensive thermal damage with postmortem carbonization.
Unique findings	Cervical vertebrae disruption; significant sinusoidal dilation in the liver.	Hyaline membranes in alveoli indicating acute respiratory distress; focal myocardial necrosis.	Prominent ischemic damage in the kidneys with tubular epithelial detachment.	Thermal coagulative necrosis obliterated most tissues; no detailed histological findings possible.	Extensive retroperitoneal hemorrhage; fibrin deposits in alveolar walls.	Diffuse cortical edema with extensive perivascular hemorrhages; inflammatory cells surrounding hepatic necrotic areas.	Subpleural emphysema; bronchial aspiration of blood.	Fragmentation of the body with remains recovered in 6 separate bags; carbonized maxilla with calcined teeth; multiple fragments of bone and soft tissue severely burned and embedded with soil and vegetation.

## 4. Discussion

The case analyses presented in this study highlight the practical difficulties encountered during postmortem examinations of victims exposed to high-energy impacts and extreme thermal conditions. Across all 8 cases, identification was accomplished using a combination of traditional forensic methods, including DNA analysis, forensic dental evaluation, visual recognition, and personal artifact examination. These findings reinforce the idea that successful identification of mass casualty events often depends on the preservation of even the smallest of individualizing details.

Our data demonstrates that despite the severity of trauma and extensive carbonization in many cases, proper identification can still be achieved using classic forensic tools. Dental prostheses (Case 3), tattoos (Case 1), and distinctive personal items, such as military tags and wedding rings (Cases 4 and 7, respectively) played a crucial role. This underscores the importance of documentation of antemortem characteristics in military and other high-risk occupations and the continued relevance of physical identifiers even in the digital age. In contrast, cases 2 and 5 required exclusion-based identification because of the complete lack of identifiable external features.

In addition, serological analysis for blood group determination was performed where biological samples were permitted, providing supplementary, yet limited information that can support the identification process in certain cases. However, 4 of the confirmed victims shared blood group A, which limits the discriminatory value of this marker in the context of multiple casualties. Furthermore, in 2 cases (3 and 8), blood group determination could not be performed because of the unavailability of suitable biological material. These findings show both the utility and limitations of blood group analysis in mass fatality incidents, emphasizing the need to interpret these results within a broader perspective.

These instances reflect the challenges posed by the absence of comparative antemortem data, stressing the necessity for more databases containing dental records, prosthetic information, and personal identifiers for individuals in such roles.

### 4.1. Limitations of the study

This study has several inherent limitations. First off, transparency and reproducibility were limited because DNA analysis was outsourced to another institution and the precise techniques used were not made available. Serological and toxicological testing were hampered in some cases by the lack of viable biological samples. Blood group analysis has limited discriminatory power which makes it less useful in situations involving multiple victims. Personal artifacts like jewelry, military tags, or tattoos were often used for identification. Although useful, these items are prone to deterioration and should be verified by genetic evidence. In some cases, microscopic analysis of tissue samples was not possible due to severe thermal damage, which limited our understanding of the causes of death and postmortem interval. Furthermore, the identification process was made more difficult by the lack of antemortem data, such as dental records or prosthetic documentation. A small sample size and the exclusion of certain contextual information because of confidentiality concerns further limited the study’s scope.

### 4.2. Future directions: smart materials and forensic innovation

The latter part of the discussion introduces emerging technologies. Inspired by the limitations observed in our casework, it is critical to frame these as potential solutions. Despite the use of standard methods, the inability to determine identity in a straightforward manner in certain cases justifies the exploration of these innovations. The integration of smart materials and novel technologies is thus proposed as a forward-looking strategy that can supplement and improve the existing forensic toolkit when traditional markers are rendered unrecognizable.

In recent years, forensic artificial intelligence (AI) tools have gained increased interest. Applications such as mandibular reconstruction and postmortem interval determination can help clarify some of the circumstances regarding victims of mass disasters and have displayed promising results. Although the potential of such tools is clear, challenges such as ethical aspects and data confidentiality remain an issue.^[8]^

Fingerprint analysis can also be refined using AI applications, in those cases where a biometrics database is available for comparison. Automatic or semi-automatic fingerprint processing can limit human bias and can potentially uncover previously undetectable patterns between samples.^[9]^

Human dentition is known to be an invaluable parameter when it comes to identifying victims of mass disasters, especially if dental treatments have been previously performed. Automatic analysis of a dental orthopantomogram (OPG) using deep neural networks proved to be a reliable method of comparing antemortem samples with postmortem ones, in cases in which they were available.^[9]^

Technological advancements and traditional investigative exploration methods continue to impact reliable identification in forensic cases, as previously shown by the analysis of such cases.^[10]^ Forensic cases also have to consider the important role played by craniofacial injuries as well as thermal damage in their determination, as per the literature.^[11,12]^ These findings align with those of studies emphasizing the criticality of dental evidence in the absence of intact physical remains.^[13]^ Traditional human dentition is referred to as the most unique biological tissue and cannot be altered, as stated in several studies by Popa et al It is beneficial in forensic odontology, especially in the reconstruction of faces after traumatic cases involving high-energy impact and extreme temperatures.^[14–17]^ In severe carbonization scenarios, dental prostheses embedded with intelligent materials such as NFC tags and aerogels have emerged as robust alternatives for preserving identity-related data.^[18]^ Neculai-Cândea et al demonstrated that Aerogel, when combined with NFC tags, preserves data integrity even under harsh environmental conditions, including temperatures up to 900°C.^[19]^ This technological advancement provides a significant advantage over traditional engraving or labeling techniques, which often fail under similar circumstances.^[20]^

Such innovations are critically important in forensics, where visual identification or DNA analysis is difficult because some structures or cells are already highly damaged.^[21]^

Popa research on palatine folds as unique identifiers also finds applications in cases requiring advanced differentiation when standard methods are insufficient.^[22–24]^ The permanence and individuality of palatine folds add another layer to the forensic toolkit, bridging the gap between odontological and anthropological approaches.^[25,26]^ The use of smart materials in forensic science has further enhanced the practice of forensic odontology.^[27]^ By embedding NFC tags in dentures using innovative sandwich techniques, it is possible to preserve critical identification data over extended periods, even in liquid or acidic environments.^[28,29]^ This is complemented by what Stefanescu et al claim: that even if physical evidence is lost, data embedded within can be retrieved.^[22]^ This is useful for forensic identification methods targeted at mass death or high-energy types of trauma.^[30,31]^

Thus, the synergy of advanced technological methods and traditional forensic practices enhances the reliability of identification processes.^[32]^ As evidenced by the detailed forensic investigations in this study, incorporating intelligent materials into forensic workflows can address the challenges posed by severe environmental and traumatic damage.^[33]^ Future studies should seek possible ways of using these technologies in wider areas of forensic and judicial research to develop uniform technical conditions for their application.^[34]^

## 5. Conclusions

The analysis of cases highlights the importance of prioritizing antemortem data documentation for high-risk occupations, adjusting forensic techniques, and utilizing personal artifacts for identification. Collaboration between military and emergency response institutions is crucial for accurate victim identification. The integration of intelligent materials like NFC tags in dental prostheses can enhance reliability and accuracy of identification processes. Further research could involve utilizing smart materials in disaster scenarios and creating global forensic regulations.

## Author contributions

**Conceptualization:** Stefan Pricop, Sorin Deacu.

**Data curation:** Stefan Pricop.

**Formal analysis:** Miruna Cristian, Sorin Deacu.

**Investigation:** Stefan Pricop, Sorin Deacu.

**Methodology:** Stefan Pricop.

**Project administration:** Miruna Cristian.

**Supervision:** Miruna Cristian.

**Validation:** Miruna Cristian, Sorin Deacu.

**Writing – original draft:** Stefan Pricop, Radu Adrian Nitu, Sorin Deacu.

**Writing – review & editing:** Miruna Cristian, Radu Adrian Nitu, Sorin Deacu.
